# Environmental versus operational drivers of drifting FAD beaching in the Western and Central Pacific Ocean

**DOI:** 10.1038/s41598-019-50364-0

**Published:** 2019-09-30

**Authors:** Lauriane Escalle, Joe Scutt Phillips, Maurice Brownjohn, Stephen Brouwer, Alex Sen Gupta, Erik Van Sebille, John Hampton, Graham Pilling

**Affiliations:** 1 Oceanic Fisheries Programme, Pacific Community (SPC), B.P. D5, 98848 Nouméa, New Caledonia; 2 The Parties to the Nauru Agreement (PNA) Office, P.O. Box, 3992 Majuro, Marshall Islands; 3 Climate Change Research Centre and ARC Centre of Excellence for Climate Extremes, University of New South Wales, Sydney, Australia; 4 Institute for Marine and Atmospheric research, Utrecht University, Utrecht, Netherlands

**Keywords:** Biodiversity, Conservation biology, Ecosystem ecology, Marine biology

## Abstract

In an effort to increase purse seine fishing efficiency for tropical tunas, over 30,000 drifting Fish Aggregating Devices (dFADs) are deployed every year by fishers in the Western and Central Pacific Ocean (WCPO). The use of dFADs also impacts ecosystems, in particular through marine pollution and dFAD beaching. This paper presents the first estimate of dFAD beaching events in the WCPO (>1300 in 2016–2017) and their distribution. Lagrangian simulations of virtual dFADs, released subject to contrasting deployment distributions, help us determine the relative importance of operational versus environmental drivers of dFADs drifting to beaching areas. The highest levels of beaching, occurring on Papua New Guinea and Solomon Islands, are likely a result of the prevailing westward oceanic circulation and subsequent local processes driving dFADs towards land. Similarly, high beaching rates in Tuvalu appear to be due to the general circulation of the WCPO. In contrast, beaching in Kiribati Gilbert Islands appear to be more strongly related to dFAD deployment strategy. These findings indicate that reducing beaching events via changes in deployment locations may be difficult. As such, management approaches combining dFAD deployment limits, the use of biodegradable dFADs, recoveries at-sea close to sensitive areas and/or beached dFAD removal should be considered.

## Introduction

Over the last few decades there has been a fundamental shift in the way tuna aggregations are tracked and subsequently caught. Oceans now contain tens of thousands of drifting Fish Aggregating Devices (dFADs) that provide accurate real time information on their locations, and an estimate of the aggregated biomass below the dFAD^1,2^. Fishers have always used the tendency of natural floating objects to aggregate fish to increase fishing efficiency, but today fishing on man-made dFADs has become a major fishing mode for purse seine fisheries worldwide^3,4^. While Ecosystem-Based Fishery Management (EBFM)^5^ calls for the reporting of ecosystem impacts and incidentally captured species in addition to that of target species, the management of dFADs has largely been concerned with the impacts on target tuna stocks and problems of relatively high bycatch rates^6–8^. Recently however, concerns have been raised about marine pollution, particularly associated with dFAD beaching (i.e. stranding in coastal areas). Such events can damage reefs and related ecosystems, and lead to ghost fishing, pollution and the loss of expensive fishing gear^9^.

The Western and Central Pacific Ocean (WCPO) contains the largest tropical tuna purse seine fishery in the world, with more than 1.8 million tonnes captured in 2017^4^, mostly composed of skipjack and yellowfin tuna, with a smaller catch of small bigeye tuna in dFAD sets. This region also has the highest number of dFAD deployments in the world, estimated at more than 30,000 per year^1,10^. The WCPO purse seine fishing grounds largely occur in the exclusive economic zones (EEZs) of Pacific Island Countries, whose coasts are lined by coral reefs. Finally, prevailing westward currents across the tropics carry dFADs deployed in the Eastern Pacific Ocean (EPO) to the west^11^. Given the high number of dFADs deployed and the relatively large number of Pacific Islands with sensitive coral reefs, the effects of lost dFADs and potential for beaching events implies that the WCPO may be the most at risk ocean basin. In this context, understanding the processes leading to beaching events is a key element in dFAD management in the WCPO^12,13^.

In principle, beaching events are influenced by (i) the density of dFADs near landmasses and (ii) local bathymetry and ocean circulation close to land. In the first case, the density of dFADs depends on both the dFAD deployment distribution and quantity and the subsequent redistribution of dFADs by regional ocean currents that can aggregate or disperse passive floating objects^14,15^. Therefore, in the absence of local effects, the number of beaching events along a coast should be proportional to the density of dFADs around that landmass, with more beaching events occurring in areas of higher dFAD density. Secondly, local currents may enhance the retention of dFADs close to islands and so lead to higher levels of local beaching. Areas with high beaching and dFAD density may therefore be linked to the dFAD deployment strategy and broad-scale ocean drift patterns. Conversely, areas with much lower or higher numbers of beaching events than expected, given the local dFAD density, are likely to be influenced by local bathymetry or circulation near the coast. Understanding these drivers of beaching events is important to effectively manage dFAD purse seine fisheries in the WCPO.

**Figure 1:**
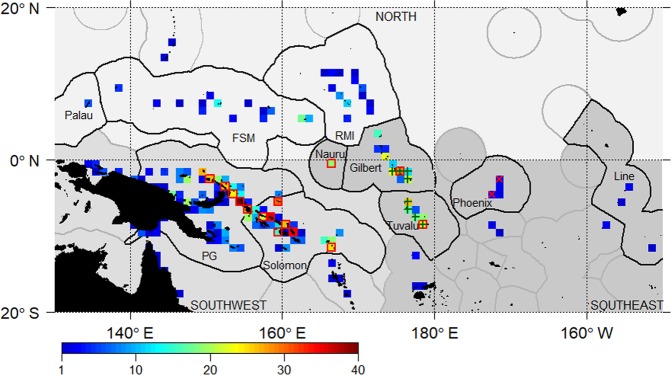
Number of beaching events (1,320 in total) per 1° grid cell across 2016 and 2017. Countries that form the Parties to the Nauru Agreement (Federated States of Micronesia, Kiribati (Gilbert, Phoenix and Line Islands), Republic of the Marshall Islands, Nauru, Palau, Papua New Guinea (PG), Solomon Islands and Tuvalu) and the three defined beaching areas (north, southwest and southeast) are indicated on the map (different shades of grey). Notable beaching cells are displayed as follows: (i) high-density cells (green plus) where beaching events are proportional to dFAD density; (ii) beaching-prone cells (red crosses) with high numbers of beaching events but low local dFAD density; and (iii) beaching-resilient (red squares) cells with low numbers of beaching events and high local dFAD density.

In order to monitor dFADs and aid management of the purse seine fishery, a programme has been implemented to track dFADs within the EEZs of the Parties to the Nauru Agreement (PNA, see Fig. 1). The availability of trajectories of over 22,000 dFADs has revealed a considerable number of probable beaching events, and in many cases their deployment and drift pattern prior to beaching^16^. However, given the high level of fine-scale variability in ocean circulation, small variations in the initial deployment location may lead to varied trajectories and final positions over time^17^. Therefore, quantifying the connectivity between areas of high beaching and the surrounding ocean in a statistically robust way would require observations from many more dFADs.

Lagrangian particle analysis offers a useful tool to supplement the observed dFAD tracks^18,19^. Using time-evolving velocity fields from a high resolution ocean circulation model, large numbers of virtual particles with float characteristics that mimic dFADs can be seeded into the simulated ocean to quantify the possible pathways of these floating objects. Such simulations have been used to understand many aspects of ocean circulation and to investigate the connectivity of passively drifting larvae^20^, nutrient flow^21^, marine plastics^22^, non-passive agents such as tuna themselves^23^, as well as dFADs^24^. In the context of dFAD beaching, Lagrangian particle simulation experiments can provide an independent estimate of the broad-scale probability distribution of connectivity between the ocean and beaching events. By seeding particles over the entire study area under differing deployment scenarios, it is possible to examine the potential source/sink dynamics of dFADs arriving in known beaching areas.

Analyses presented in this paper are the first to estimate the distribution of dFAD beaching events from the largest tropical tuna purse seine fishery in the world. dFAD beaching events and their corresponding deployment locations were analysed using data from 22,000 dFADs deployed in the WCPO in 2016–2017, as well as Lagrangian simulations of over 1.5 million virtual dFADs. Using the trajectories of both real beached dFADs and simulated particles from Lagrangian simulation experiments, the connectivity between these observed beaching areas and dFAD deployment source locations was quantified.

## Methods

### dFAD tracking data

In order to quantify and manage the number of dFADs deployed in and drifting through the EEZs of PNA members (Federated States of Micronesia, Kiribati, Republic of the Marshall Islands, Nauru, Palau, Papua New Guinea, Solomon Islands and Tuvalu), a dFAD-tracking programme was initiated in January 2016. This programme requires fishing companies to report data from satellite buoys deployed on dFADs to the PNA via the satellite service provider. These data consist of a location and time stamp recorded periodically by the dFAD buoy. Here, these data are used to investigate beaching events and the associated deployments and drift tracks between 1 January 2016 and 31 December 2017. Transmission frequency (usually every hour) may vary over time due to fishers setting different transmission ‘modes’. For example lower frequencies are typically used when dFADs drift away from main fishing areas or during the WCPO dFAD closure period. This annual dFAD closure corresponds to a three to four month period (July to September, extending to October for some vessels), where all dFAD-related activities (deployment, setting and servicing) are prohibited^25^. However, there is no obligation to remove dFADs from the water and beaching can still occur. Transmissions start when the buoy is activated, which can be a few hours to several days before deployment, and continue until deactivation (e.g. dFAD lost, retrieved, beached or outside the productive area that each vessel operates in).

The trajectories of 26,921 buoys with 13.6 million transmissions were assessed. This provides location data for when dFADs were drifting at sea, but may also include some data from when the dFAD is onboard a vessel either before deployment or when dFADs were recovered at sea. An initial data cleaning process was undertaken to remove buoys activated for only short periods, buoys with transmissions from only single positions, double transmissions or consecutive transmissions corresponding to unrealistic drift speeds. Transmissions when buoys were on-board a vessel were then removed. Positions were classified into “at-sea” or “on-board” following the method developed by Maufroy *et al*.^26^. This method uses a Random Forest model^27,28^ based on: time interval between consecutive transmissions; speed; acceleration; heading change; and distance from major port. Additional corrections were undertaken to reduce segmentation rates (i.e. remove isolated “at-sea” or “on-board” positions)^26^. Each dFAD track then consisted of one (67% of the buoys) or several (2–14 segments in 33% of the buoys) segments of drift positions. A buoy trajectory may represent a single buoy deployed on several dFADs, i.e. floating platforms with submerged appendages, following separate recovery and deployment events. For each segment, deployment position was estimated as the first “at-sea” position. Note that some dFAD trajectories were modified on instruction by fishing companies prior to submission so that only the part of the track within PNA waters remained. This modification of the data creates a bias in the identification of deployment positions (5.6% of deployments were found at the boundary of a PNA member EEZ^16^), and beaching events. However, the bias is probably small given that the PNA waters are the main dFAD purse seine fishing grounds in the WCPO and fishers routinely deactivate dFADs drifting out of the main fishing areas.

Following the method of Maufroy *et al*.^26^ beaching events were assumed when dFADs had (i) the last recorded position within 10 km of shore (excluding 240 dFADs with positions located at less than 10 km from major ports); and (ii) at least the 3 last transmissions from the same location. Coastal cells (1 × 1°) with at least one beaching event were classified as beaching cells (Fig. 1). The spatial distribution of dFAD deployments was investigated using smooth kernel densities^29^ (‘kde2d’ and ‘filled.contour’ functions in R package MASS^30^) and deployment hotspots were defined as an area with number of deployments per grid cell exceeding the 95^th^ percentile based on all 1° cells. Beaching events and corresponding deployments were studied by pre-defined areas of the WCPO within or outside deployment hotspots and by year/quarter.

### Identification of notable beaching locations

**Figure 2:**
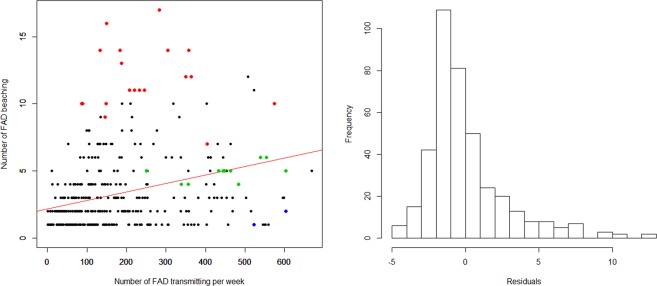
Regression between number of beaching events and local dFAD density per 1° grid cell with a seasonal factor and histogram of residuals from the model. R^2^ = 0.10 for the linear model; p-value < 0.001. Coloured dots represent notable beaching cells: (i) high density cells (green) with high number of beaching events and high local dFAD density; (ii) sensitive cells (red) with high number of beaching events but low local dFAD density; and (iii) resilient cells (blue) cells with low number of beaching events and high local dFAD density.

To examine individual beaching locations, the relationship between the number of beaching events (across 156 beaching cells) and observed standardised (one transmission per week) local dFAD density per 1° beaching cell was investigated. Outliers from the linear regressions (Fig. 2) between beaching events and local dFAD density were used to classify “notable beaching cells” with specific characteristics.

### Lagrangian particle simulations

To examine the connectivity between the beaching cells and ocean area, the drift trajectories of dFADs were simulated over the whole study area (i.e. the WCPO tuna purse seine fishing grounds: 130°E–140°W and 15°S–10°N) using a passive Lagrangian particle simulator, Parcels^31^. Particles were advected (offline) in velocity fields averaged over the top 50 m of ocean from the 1/12° HYCOM + NCODA Global Analysis with a daily time step (http://hycom.org). This 50 m depth represents the median depth of attached dFAD appendages used as drogues (based on information recorded by on-board scientific observers from 2011–2016 for the WCPO^32^).

Two Lagrangian simulation experiments were explored based either on a deployment distribution that was uniform across the study region or on a non-uniform distribution representative of real deployments by fishers. The *uniform deployment scenario* was carried out to examine the role of large-scale ocean currents in determining the connectivity between ocean regions and identified beaching areas. In this simulation, particles were randomly released across the entire study area with a mean density of five particles per 1° cell. In the *observed deployment scenario*, particles were released across the entire study area, but with a probability equal to the distribution estimated from observed dFAD deployments (i.e. the smooth kernel density of deployment described above). In both cases, new particles were seeded every seven days at a rate of over 11,000 particles per week. Simulation experiments extended from July 1^st^ 2015 until December 31^st^ 2017, with the initial 6 months (average life time of a dFAD^16^) treated as a spin up period. The analysis period was 2016–2017. In total, over 1.5 million particles representing virtual dFADs were released for each experiment. Each particle was assumed to drift for a maximum of 1 year to avoid over-accumulation of particles.

In order to test the fidelity of the simulations in estimating dFAD aggregation in coastal areas, the density of observed and simulated dFADs were compared across 502 1° cells around landmasses. Subsets of the particle trajectories that ended or passed through any of the observed beaching cells (156) were then extracted to quantify the abundance and connectivity of particles entering these areas. Given the 1° resolution used and the occurrence of local processes, it was not assumed that trajectories ended when the particle entered a beaching cell. For each beaching cell, the trajectory of any particle that entered the cell during the simulation was considered a “beaching trajectory”. If particles passed through multiple beaching cells, a separate beaching trajectory was taken for each cell and was treated as independent.

### Connectivity of beaching events

For both observed and simulated beaching trajectories, the connectivity between beaching cells and ocean areas was quantified. All observed and simulated beaching trajectories were compiled and the position of dFADs or simulated particles were determined for several periods prior to beaching (<1 month, 1–3 months, 3–6 months, 6–9 months, >9 months). To study the connectivity between wide regions of the WCPO, beaching cells were grouped into broader regions of neighbouring EEZs with similar beaching patterns. Similarly, positions of dFADs at increasing periods prior beaching were grouped into broader regions, reflecting the deployment hotspots.

## Results

### Observed dFAD beaching and deployment hotspots

The cleaned dataset consisted of 22,620 observed dFAD trajectories (84% of the original dataset) split into 32,665 at-sea drift trajectories separated by periods on-board a vessel (15,455 dFADs had a single, continuous drift track) (see Escalle *et al*.^16^ for a detailed overview of the dFADs tracking dataset). Of these, we estimated that 5.8% of all dFAD trajectories (1,320) ultimately beached. The largest number of beaching events were in the EEZs of Papua New Guinea (483), Solomon Islands (379), Kiribati Gilbert Islands (155) and Tuvalu (117) (Fig. 1). High seasonal variability in beaching was detected. In the 1^st^ quarter, Nauru had the highest beaching density per 1° grid cell (10 beaching events). During the 2^nd^ quarter, a relatively high number of beaching events occurred in Papua New Guinea (142), as well as during the 4^th^ quarter in Solomon Islands (110) and Tuvalu (49). Note that in Kiribati Gilbert Islands, beaching events remained constant during the first three quarters (around 40 per quarter).

In view of the distribution of beaching events (Fig. 1), three “beaching regions” were defined: (i) the southwest area comprising the EEZs of Papua New Guinea and Solomon Islands (and any other areas west of 175°E), with the highest number of beaching events per single cell and per EEZ; (ii) the southeast area comprising mostly the EEZs of Nauru, Kiribati Gilbert Islands and Tuvalu, with relatively high numbers of beaching events by cell; and (iii) the north area comprising mostly Federated States of Micronesia and Republic of the Marshall Islands EEZs, which presented a lower number of beaching events.

**Figure 3:**
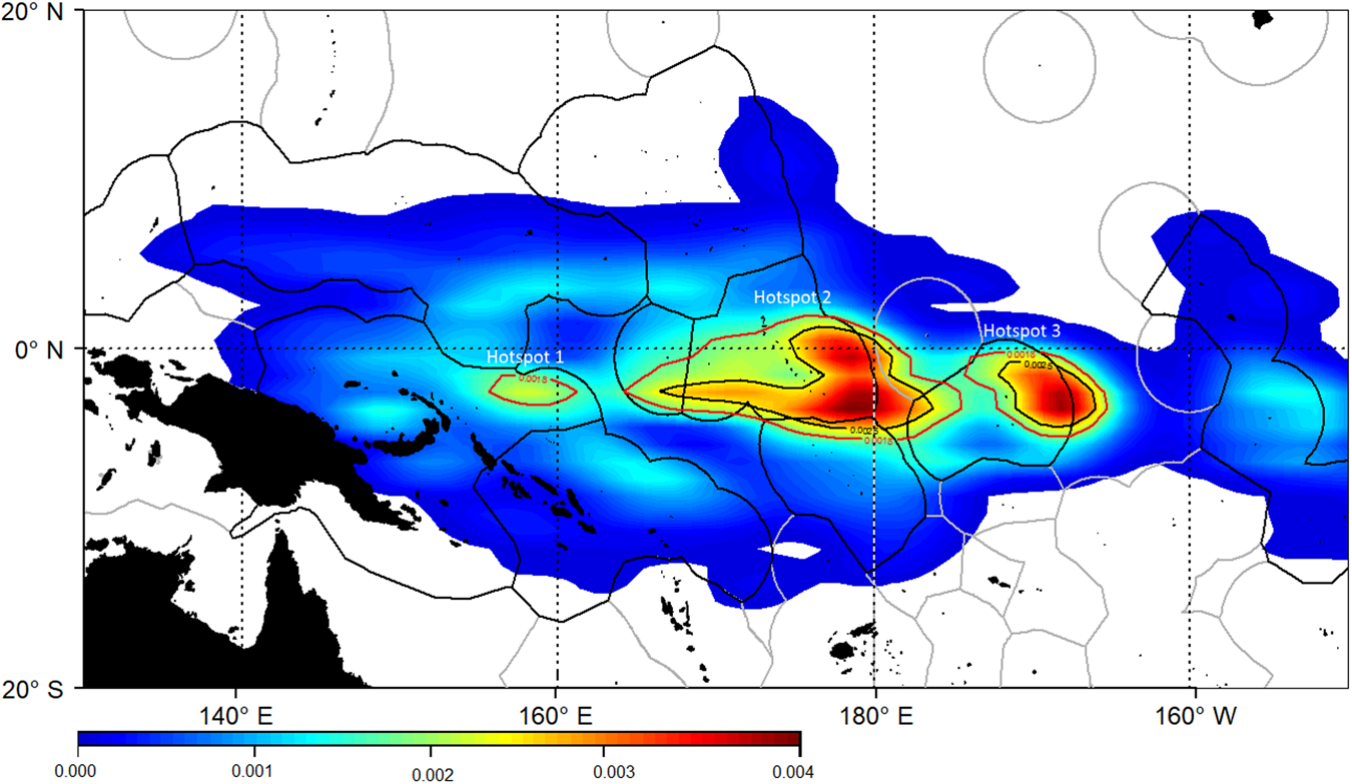
Smooth kernel densities of dFAD deployment locations, estimated from observed trajectories. Red and black lines correspond to the 95^th^ and 98^th^ percentiles.

Smooth kernel density maps of deployment positions for all observed dFADs (Fig. 3) show three main deployment hotspots, based on 95^th^ percentile of the data: (i) east of the Papua New Guinea EEZ (Hotspot 1); (ii) a large hotspot in the centre of the WCPO mostly covering Kiribati Gilbert Islands, Nauru, north of Tuvalu and international waters (Hotspot 2); and (iii) east of Kiribati Phoenix Islands (Hotspot 3). Deployments in the rest of the WCPO were divided into three regions, which mirrored the classification of the beaching regions: (i) southwest area (<0°N and <175°E); (ii) southeast area (<0°N and > = 175°E); and (iii) north area (>0°N). These six deployment regions were then used in the connectivity analysis to investigate the link between observed deployment and beaching regions.

### Notable beaching locations

**Figure 4:**
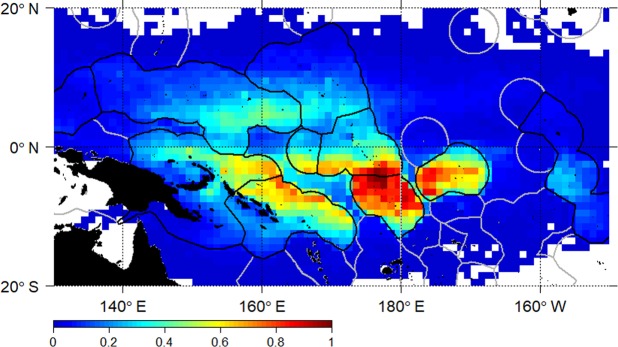
Normalised observed dFAD density by 1° grid cell derived from dFADs transmitting position at least weekly. Scale corresponds to cells having a least one dFAD transmitting for a week, and a maximum of 6,670 dFADs transmitting at least weekly (same dFAD may be counted several times in different weeks and cells).

Observed normalised local dFAD densities (Fig. 4) were used to identify beaching locations with particularly high or low numbers of beaching events relative to their local dFAD density. A linear model of the number of dFAD beaching events and local dFAD density per 1° grid cell (Fig. 2), indicates a general increase in beaching events with local dFAD density, although with a relatively low coefficient of determination (R^2^ = 0.10; p-value < 0.001). However, it is clear that some cells have particularly high or low beaching events for a given local dFAD density. Residuals from the linear model were used to identify three types of notable beaching cells: (i) “high density cells”, where beaching events are proportional to dFAD density (selecting events where residuals are within the center 10^th^ percentile of the dFAD distribution and the number of beaching events above the 80^th^ percentile); (ii) “beaching prone cells”, with higher number of beaching events relative to the local density (i.e. residuals above the 90^th^ percentile); and (iii) “beaching resilient cells”, with a low number of beaching events and a high local dFAD density (i.e. residuals below the 10^th^ percentile) (Fig. 1, Table 1). High density cells were identified in the southeast area (Kiribati Gilbert Islands and Tuvalu) in quarters 1–3. Beaching prone cells were mostly found in the southwest area (Papua New Guinea and Solomon Islands) in quarters 2–4, but also some in the southeast area (one cell in Nauru in quarter 1, two cells in Kiribati Gilbert Islands in quarter 3 and one and two cells in Tuvalu in quarter 4) (Fig. 1). Finally, only two beaching resilient cells were found, both located in the southeast area (Kiribati Phoenix Islands) in quarter 1.

**Table 1:** Number of notable beaching cells from the observed dataset per beaching region of the WCPO.

Beaching region	No. of high density beaching cells	No. of beaching prone cells	No. of beaching resilient cells	No. of beaching events
North	0	0	0	137
Southwest	0	15	0	910
Southeast	11	4	2	319

High density cells = high number of beaching events and high local dFAD density (residuals from the linear model between number of beaching events and number of dFADs per 1° cell within the center 10^th^ percentile of the distribution and number of beaching above the 80^th^ percentile); beaching prone cells = significantly higher number of beaching events but low local dFAD density (residuals above the 90^th^ percentile); and beaching resilient cells = low number of beaching events and high local dFAD density (residuals below the 10^th^ percentile).

### Simulated dFADs

Based on the *observed deployment scenario*, a significant correlation was found between the standardised density of virtual dFADs and observed dFADs across coastal cells (Pearson correlation coefficient = 0.83), with the greatest error for coastal cells with zero or low observed density (see Figures in Supplementary Material). The associated correlation from the *uniform distribution scenario* across the entire study area was considerably poorer (Pearson correlation coefficient = 0.53), highlighting the influence of deployment location in determining the observed density of real dFADs in these coastal cells.

**Figure 5:**
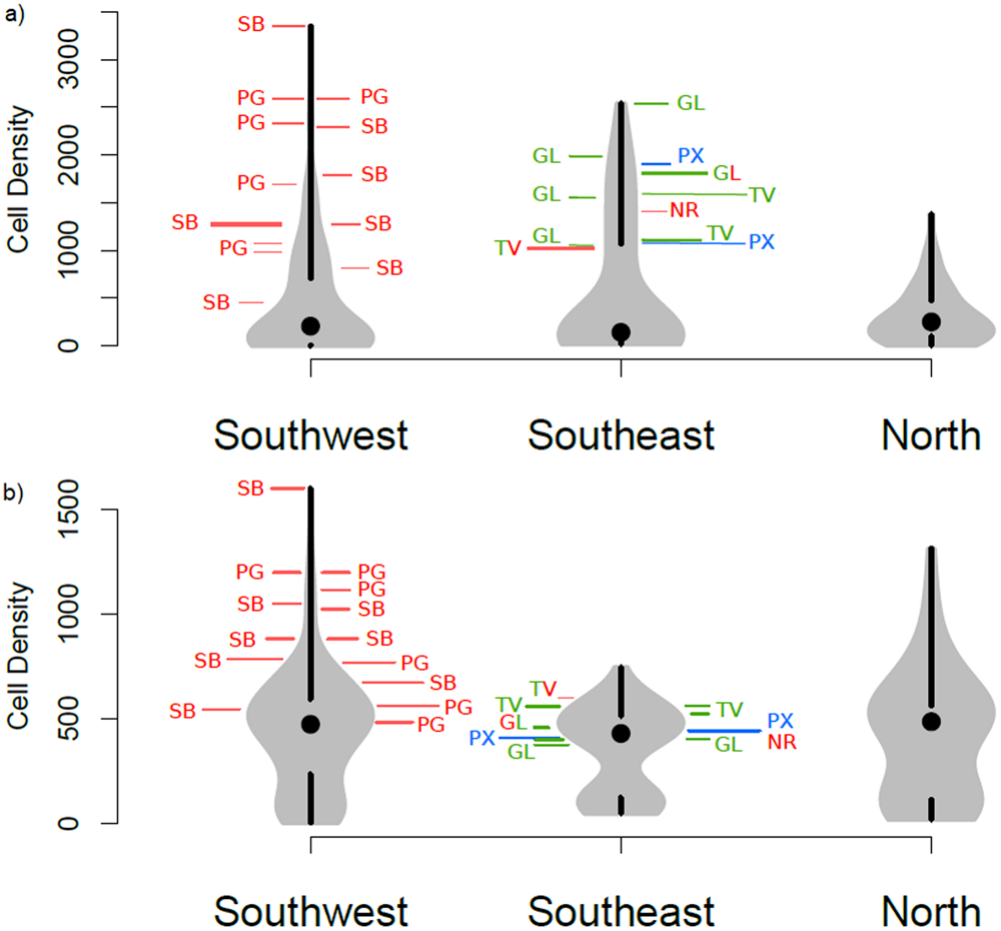
Violin distribution plots of mean daily number of simulated particles present in each coastal cell, separated by beaching region, for the (**a**) *observed deployment* and (**b**) *uniform deployment scenarios*. Black dots show median values, with black lines marking upper and lower quartiles. Overlaid are the positions of notable beaching cells (SB = Solomon Islands, PG = Papua New Guinea, NR = Nauru, TV = Tuvalu, GL = Kiribati Gilbert Islands, PX = Kiribati Phoenix Islands), and coloured to indicate classification as (i) high-density (green); (ii) beaching-prone (red); and (iii) beaching-resilient (blue). Two colours indicate two different cells categorisations, depending on season.

To explore the effect of deployment location on beaching, the particle density in the classified, notable beaching cells was superimposed over the distribution of particle density in all coastal cells (Fig. 5). Based on the *observed deployment scenario*, distributions were positively skewed in all regions, with the majority of coastal cells having a relatively lower density of virtual dFADs, and a smaller number of cells having a very high density (Fig. 5). Coastal cells in the north beaching area generally had low simulated particle density. All simulated particle densities from notable beaching cells were in the top half of the distribution of both southern beaching regions where they occurred, and within the top quartile for the majority. High-density and beaching-resilient cells were all in the top quartile of coastal cell densities in the southeast beaching region, with one high-density beaching cell in Kiribati Gilbert Islands having the highest simulated dFAD density of any cell in the southeast region. For beaching-prone cells, positions in the distribution were more varied, though one prone cell in the Solomon Islands had the highest density of any coastal cell in the whole domain.

Using the *uniform deployment scenario*, in which particles were seeded uniformly throughout the study area, the distribution of simulated particle density for coastal cells was very different. Maximum density values were lower, though medians were higher, with a bimodal distribution for all beaching regions. Under this scenario, most high-density and beaching-resilient cells were located closer to the centre of the distribution around the median value. However, high-density cells in Tuvalu remained in the upper quartile of coastal cell densities in the southeast region, though absolute density values remained lower than under the *observed deployment scenario* in some cases. Beaching-prone cells were similarly spread across the top half of the distribution in all but one case, though absolute densities for some cells were again lower than under the *observed deployment scenario* (two to three times more simulated particles detected).

### Connectivity between areas

**Figure 6:**
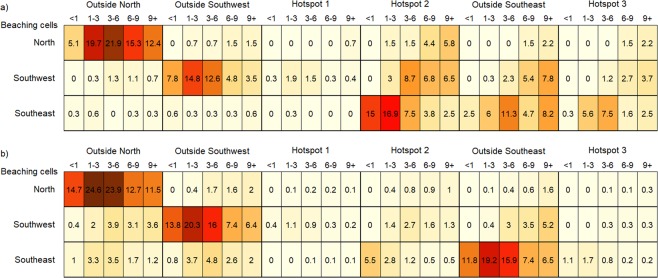
Percentage connectivity matrix of observed dFAD beaching by beaching areas against deployment areas and separated by drift time in months. Cells are coloured by proportion of dFADs and simulated particles arriving in each beaching zone by drift-time.

**Figure 7:**
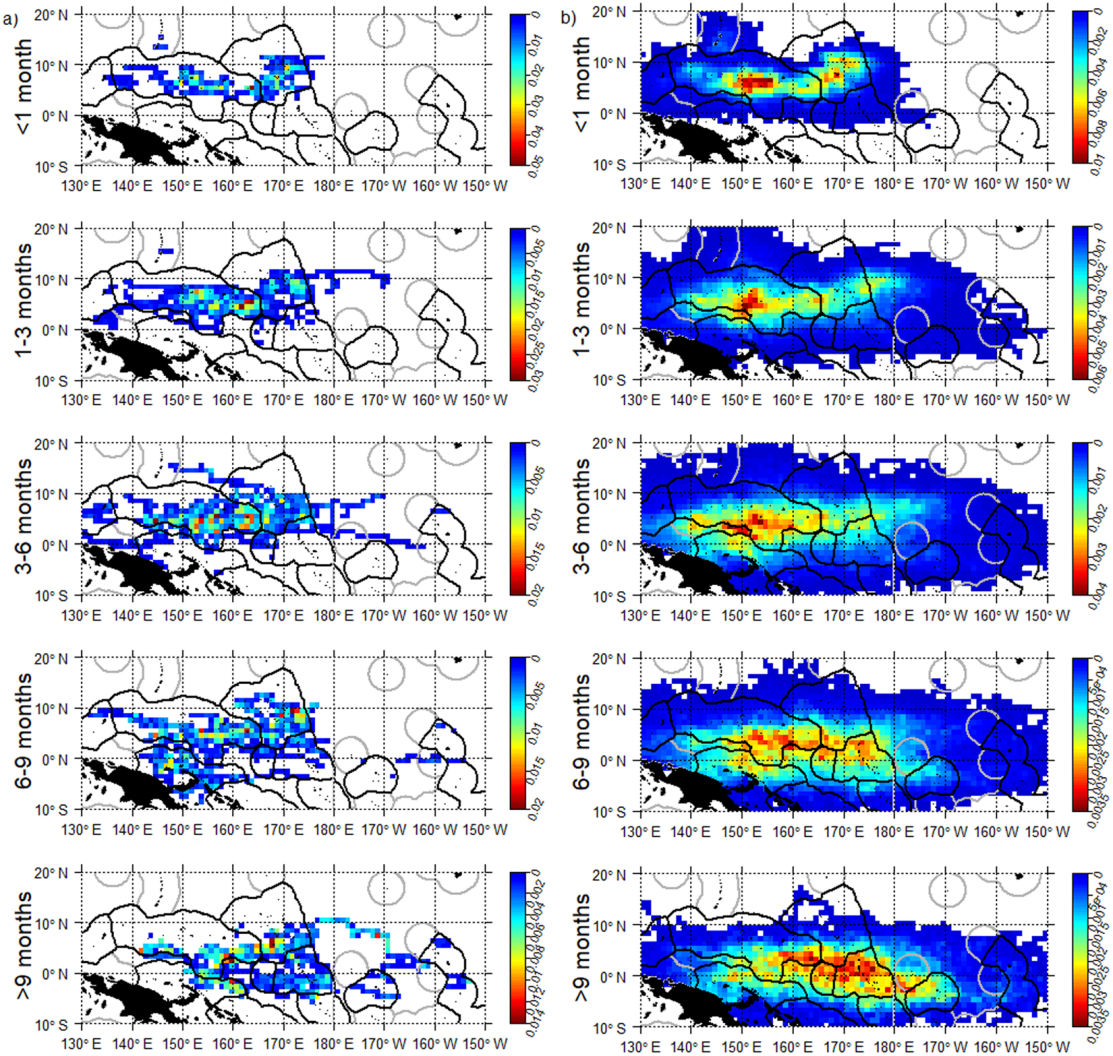
Spatial probability density for the northern region of all (**a**) observed dFADs that ultimately beached and (**b**) simulated (from the *observed deployment scenario*) dFADs entering beaching cells during five drifting-time bins prior to beaching (rows).

**Figure 8:**
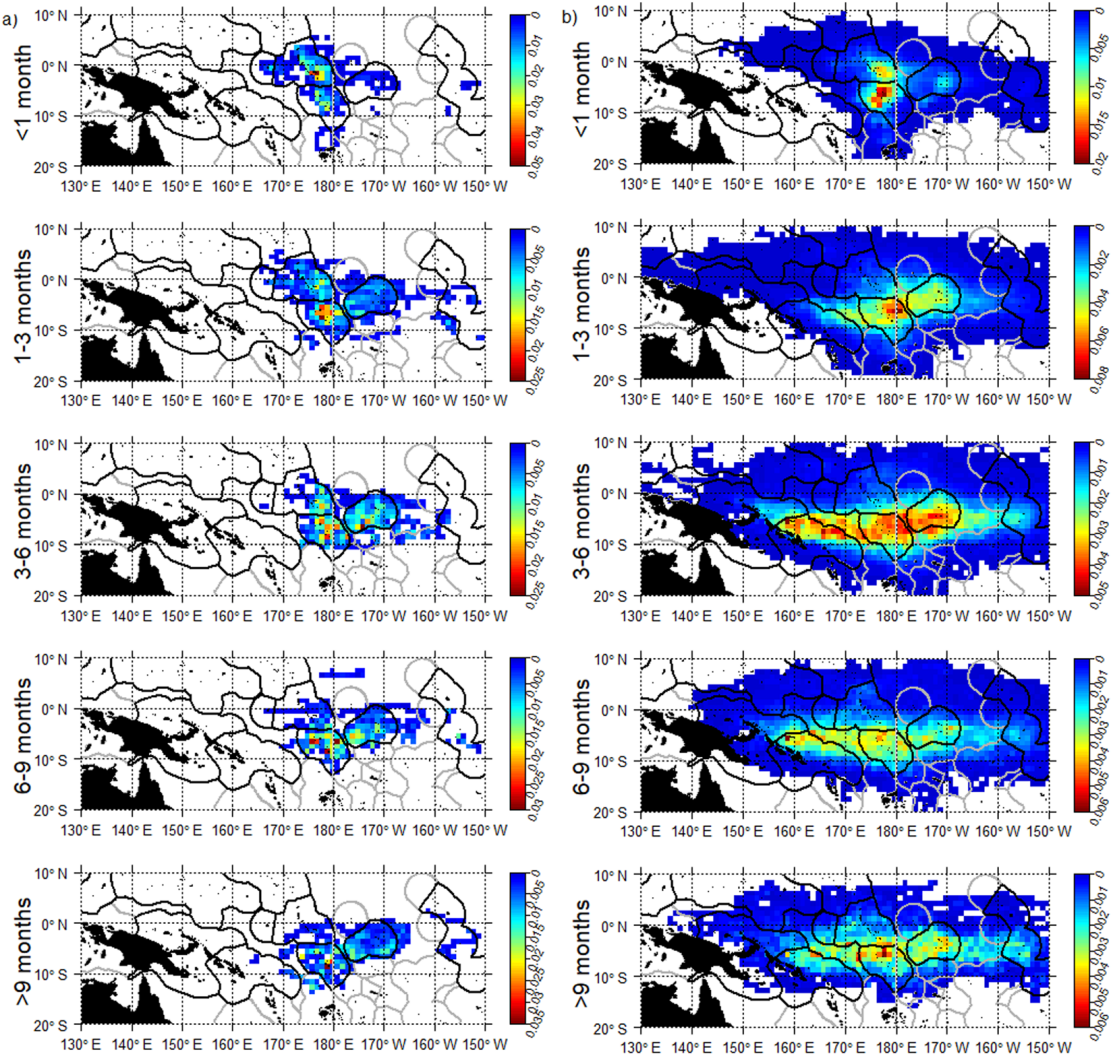
Spatial probability density for the southeast region of all (**a**) observed dFADs that ultimately beached and (**b**) simulated (from the *observed deployment scenario*) dFADs entering beaching cells during five drifting-time bins prior to beaching (rows).

**Figure 9:**
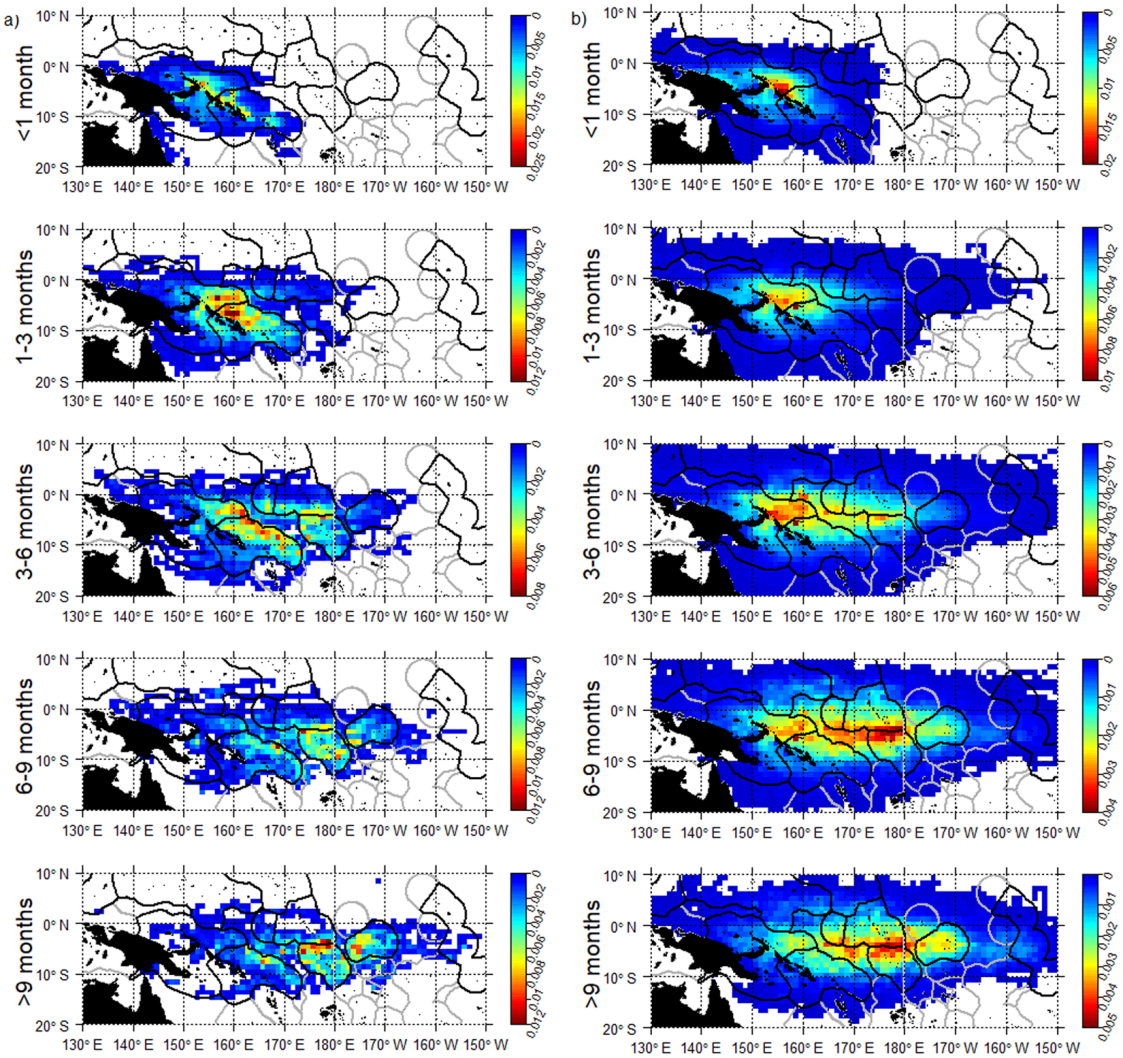
Spatial probability density for the southwest region of all (**a**) observed dFADs that ultimately beached and (**b**) simulated (from the *observed deployment scenario*) dFADs entering beaching cells during five drifting-time bins prior to beaching (rows).

Based on the observed trajectories most dFADs beached in the southern (or northern for short drift-times) hemisphere were deployed in the same hemisphere (Fig. 6). Notable beaching cells were not identified in the north area, and deployments for beaching events did not display a strong regional pattern and were distributed across the north area (Figs 6 and 7a). A connectivity analysis using simulated trajectories from the *observed deployment scenario* significantly increased the sample size of potential beaching trajectories. This analysis showed that a large proportion of beached dFADs in the north whose drift-times were longer than 6 months were deployed in the southeast area (Fig. 7b). Conversely, most beached dFADs in the southeast area were deployed in hotspot 2 with drifting times of less than 3 months, or from hotspot 3 with drift times of 3–6 months (Figs 6 and 8). Some dFADs beaching in this area are likely to have been deployed outside of these hotspots, but still within the southeast region (Fig. 8). Generally, most beached dFADs that had drifted for more than 6 months were deployed outside the hotspots dFAD deployment zones and were generally situated further to the east, as far as Kiribati Line Islands EEZ for drift-times over 9 months (Fig. 8a). This was also evident for the simulated dFAD trajectories (Fig. 8b), although the simulated probability distribution also extends to the west. Finally, beaching dFADs in the southwest area were deployed throughout the WCPO, but mostly in the southern hemisphere (Fig. 6). Most of the dFADs that beached in the southwest area were deployed in the same region (outside the hotspot) with drift times of less than 6 months, with some also deployed in the southeast area (hotspot 2 or outside any hotspot) with longer drift times (Figs 6 and 9). In most cases, both observed and simulated beaching trajectories came from the east (Fig. 9).

## Discussion

This analysis is the first to estimate the distribution of dFAD beaching events (1320) from the largest tropical tuna purse seine fishery in the world. A combination of 22,620 observed dFAD trajectories, alongside Lagrangian simulations of over 1.5 million virtual dFADs were used to examine how large-scale ocean circulation, small-scale local processes, and fisher deployment affect dFAD beaching in coastal regions within the WCPO. Beaching in Papua New Guinea and the Solomon Islands appears to be strongly tied to both large-scale ocean circulation and local processes. In Tuvalu, beaching appears chiefly linked to a high density of dFADs aggregated by large-scale oceanic circulation. The Federated States of Micronesia and Republic of the Marshall Islands experienced relatively low levels of beaching, from dFADs deployed in the northern hemisphere and influenced by ocean circulation. In contrast, in Kiribati Gilbert Islands, dFAD deployment drivers appeared to influence high-density driven beaching rates in this region.

### Occurrence of beaching

dFAD beaching contributes to pollution in coastal areas, since most components in the dFADs used in the WCPO consist of plastic, metal and electronics^33^. dFADs are generally compiled of a raft with, on average, 50 m submerged structure^32^ consisting of old purse seine nets and/or ropes. This design is particularly damaging to coral reefs when the submerged structure catches on rough coral structure. If large mesh netting is used, ghost fishing may also occurs^9^. However, quantitative studies on the impacts of beached dFADs on these habitats are still lacking.

In this study, 1320 beaching events (5.8% of all trajectories) were estimated within the WCPO over two years. In the Atlantic and Indian oceans, beaching rates were almost double this (9–10% per ocean, i.e. ~8700 dFADs in both ocean over 2007–2015^26,34^). This difference may simply be due to the greater coastline, relative to ocean area in the Indian and Atlantic basins, and strong currents transporting dFADs toward them^26,35,36^. However, it is important to note that beaching events outside PNA waters were not included in this study. Nevertheless, given the dFAD deployment rate of two to three times higher in the WCPO compared to other regions^1,10^, the incidence of beaching estimated in this study remains a significant impact on the local environment.

### Drivers of dFAD aggregation in beaching cells

The Lagrangian analysis used in this study provided a much larger sample size of virtual dFADs than available observations to explore connectivity to beaching areas. Different simulated deployment scenarios allowed separation of the likely drivers of dFAD aggregation in beaching cells, either deployment strategy (*observed deployment*) or general ocean circulation (*uniform deployment*) (Fig. 5).

In the case of beaching prone cells, which occur almost exclusively in the southwest area (Papua New Guinea and Solomon Islands), relatively high local densities of simulated dFADs emerge under both *observed* and *uniform deployment scenarios*. It appears that these cells are both (i) natural dFAD sinks, accumulating dFADs drifting from across the WCPO conveyed by a general westward ocean circulation^11^; and (ii) prone to high levels of beaching from local processes driving floating objects toward the coast. However, the observed dFAD deployment strategy still increased the likelihood of dFADs arriving at beaching cells for these locations.

The two cells in Kiribati Phoenix Islands classified as beaching-resilient appear to have high densities of dFADs chiefly because of their proximity to the large deployment hotspot in the centre of the WCPO. The low number of observed beaching events suggests that local bathymetric effects reduce the occurrence of dFADs accumulating near the coastlines of these very small islands, in contrast to the beaching prone cells of the southwest area where landmasses are larger.

For those beaching cells classified as high-density (southeast only), there was a clear difference in drivers of dFAD aggregation between EEZs. In Tuvalu, the high density of dFADs in beaching cells appears to be due to convergence effects of large-scale ocean circulation. These cells have relatively higher dFAD densities than other coastal cells regardless of simulated deployment scenario, although the *observed deployment* intensified the aggregations in these cells, two to three times higher than in the *uniform deployment scenario*. In Kiribati Gilbert Islands, however, there was a clearer effect of deployment strategy on dFAD aggregation in beaching cells. Under the *observed deployment scenario*, these cells had a simulated dFAD density five to six times greater than under the *uniform deployment scenario*, under which density was comparable to any other coastal cell in the region. As such, the high density of dFADs, and therefore beaching, in these cells of Kiribati Gilbert Islands appears to be a direct consequence of dFAD deployment strategy.

### Assessing connectivity using observed and simulated beaching trajectories

Broad-scale connectivity between beaching areas and deployment zones, based on both observed and simulated dFADs were comparable. In particular, there was limited cross-equatorial connection for dFADs. In the north area, there was no dominant direction for dFAD movement. By comparison, south of the equator, most dFADs moved westward, with dFADs that beached in the southwest primarily originating from the southeast. A notable difference was that simulated dFADs drifted both eastwards and westwards prior to arrival in beaching cells in the southeast area, compared to the generally westward direction seen in the observed dFAD beaching events.

### Data limitations of observed beaching events

It is likely that we have underestimated beaching events in non-PNA EEZs, given there were few data from outside PNA waters. Similarly, non-monitored dFADs (i.e. those with lost or deactivated buoys)^16,37^ are not accounted for. However, considering that PNA waters and the high sea pockets between their EEZs are areas with the highest dFAD densities^16^, the number of beaching events outside PNA waters may be relatively low. This was corroborated by the simulation experiment using the observed deployment distribution, where most coastal cells outside PNA waters fell into the lower part of all coastal cell density distributions (except two cells in Indonesia). The simulations also suggest a high potential for non-monitored and deactivated dFADs to reach known beaching cells in the southwest area, indicating the potential of these zones to be sinks or high throughput areas of drifting dFADs.

The findings in this paper reflect beaching conditions under specific oceanographic conditions. In particular, early 2016 corresponded to the decay phase of a strong El Niño, which was followed by neutral conditions throughout 2017. Beaching patterns and connectivity are likely to change under different ENSO phases. The prevailing westward currents tend to be stronger during La Niña compared to neutral conditions. In contrast, during El Niño the equatorial divergence of particles is weaker, with the surface equatorial circulation even running eastward, during strong El Niño conditions^38^.

Finally, dFAD designs, particularly the depth of underwater structure, may influence beaching events through changes in drift speed and direction and would also result in different impacts on habitat and species. While dFADs do have various designs in the WCPO, this information regarding dFAD design was not available for observed dFADs used in this study. Sensitivity analyses of potential beaching, through simulation of assumed varying appendage depth, could be undertaken to examine this uncertainty in more detail^39^.

### Implications of results for management

Current tuna Regional Fishery Management Organisation (tRFMO) discussions regarding approaches to mitigate dFAD loss, marine debris and beaching have included dFAD recovery prior to beaching in sensitive areas^40^, beach cleaning and/or the use of biodegradable and non-entangling dFADs^41^. While this last approach could be considered the most feasible at large spatial scales, and would reduce marine pollution and entanglement of sensitive species, these dFADs may still have a physical impact on fragile habitats such as coral reefs. The damage to reefs may be reduced for dFADs that have drifted over long periods (i.e. >6months), as biodegradable materials begin to disintegrate. However, this is unlikely to be effective in the southeast area where beaching usually occurs after short drift times. In the Indian Ocean, one Spanish fishing fleet (Organization of Associated Producers of Large Freezer Tuna Freezers OPAGAC; 15 purse seiners), in collaboration with local NGOs, implemented a programme in September 2016 to recover dFADs in the Seychelles EEZ when they drifted close to environmentally sensitive areas^40^. Such a programme would be more complicated for the WCPO given (i) the large geographic spread of dFADs; (ii) the number of small remote islands; (iii) the size of the purse seine fleet; and (iv) the number of dFADs deployed. Switching to a different management regime or designing specific measures to limit marine pollution and beaching may be more appropriate for this region. A requirement that dFADs should report their positions, both inside and outside PNA waters, would also aid better estimation of total beaching events and assist in locating dFADs already beached for potential retrieval.

WCPO-wide or region-specific deployment limits should also be considered as potential mitigation measures against negative impacts of dFAD use such as beaching. Patterns of beaching and connectivity identified in this study varied depending on region and deployment zone. Hence, overall limits would be required for effective mitigation in areas where beaching is influenced by ocean circulation (Papua New Guinea, Solomon Islands, Tuvalu and northern area’s EEZ). In contrast, a reduction in deployments within the identified hotspot zones could reduce the number of beaching events for areas where deployment strategies appeared to influence high dFAD densities (i.e. Kiribati Gilbert Islands). However, future variation in dFADs spatial deployments should be monitored and further investigations would be needed before considering spatially explicit restrictions of dFAD deployment, including an assessment of the economic impact of restrictions on dFAD deployment on both the fishing industry and Small Island Developing States.

## Conclusion

Overall, this study identified lower beaching rates compared to other oceans. The southwest tropical region, including Papua New Guinea and the Solomon Islands, had the highest number of beaching events in the WCPO. This appears to be caused by oceanographic processes driving floating objects west and then onto coastlines, irrespective of the deployment pattern. The southeast region is the main purse seine fishing area and a hotspot of dFAD deployments, which contributes to relatively high beaching rates in this region. In the Tuvalu EEZ, beaching events are linked to the large-scale circulation, while high beaching in Kiribati Gilbert Islands EEZ are more likely affected by deployment distribution. Therefore, with the exception of the Kiribati Gilbert Islands, general beaching mitigation measures may be difficult. Beaching impacts may be reduced, however, through a suite of measures including: limiting the total number of dFAD deployments; the use of biodegradable dFADs; dFAD recoveries at sea before they reach sensitive areas; and shoreline cleaning programmes.

## Supplementary information

Supplementary materials

Supplementary Dataset 1

## Data Availability

The aggregated deployment and beaching positions per 1° square of observed dFAD are available in the Supplementary Material. The Parcels package and code used to run the Lagrangian simulation experiments are freely available at https://github.com/OceanParcels/PNA_dFAD_Beaching.
