# Supplementary material for: Environmental versus operational drivers of drifting FAD beaching in the Western and Central Pacific Ocean
Source: Sci Rep. 2019 Sep 30;9:14005. doi: 10.1038/s41598-019-50364-0 (PMC6768996; doi:10.1038/s41598-019-50364-0)
Supplement: Supplementary file 1 — Supplementary materials [file 41598_2019_50364_MOESM1_ESM.docx]

**Supplementary materials**

**Environmental versus operational drivers of drifting FAD beaching in the Western and Central Pacific Ocean**

ESCALLE Lauriane, SCUTT PHILLIPS Joe, BROWNJOHN Maurice, BROUWER Stephen, SEN GUPTA Alex, VAN SEBILLE Erik, HAMPTON John, PILLING Graham


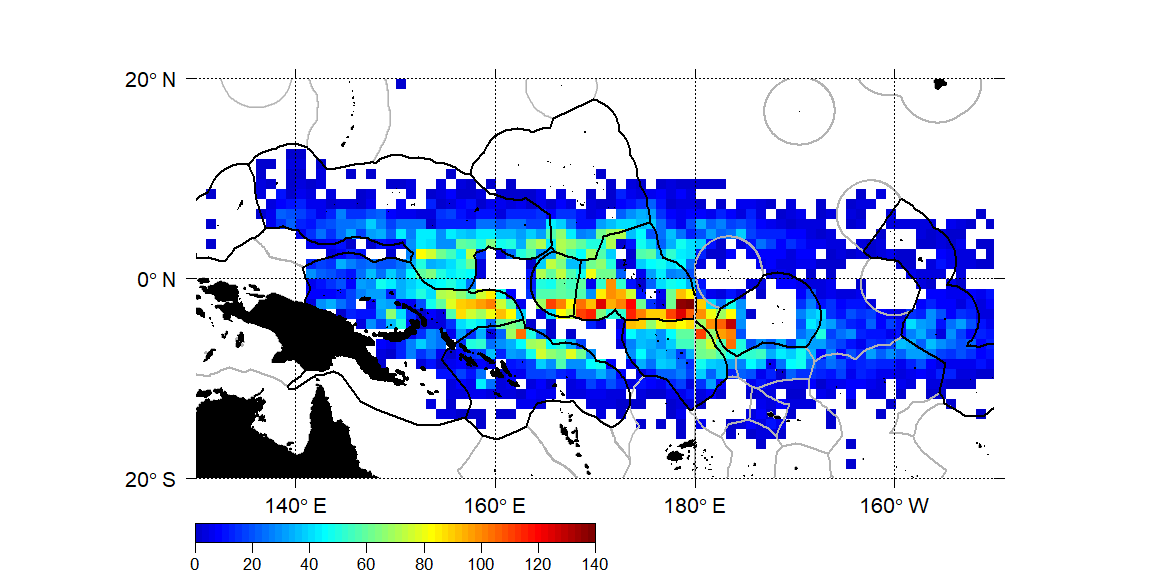


**Supplementary Figure S1.** Number of dFAD purse seine sets per 1° square from the operational logsheet data in 2016–2017 in the Western and Central Pacific Ocean. Economic Exclusive Zones of Parties to the Nauru Agreement (PNA) countries (Federates states of Micronesia, Kiribati, Republic of the Marshall Islands, Nauru, Palau, Papua New Guinea, Solomon Islands and Tuvalu) are indicated with a black line.


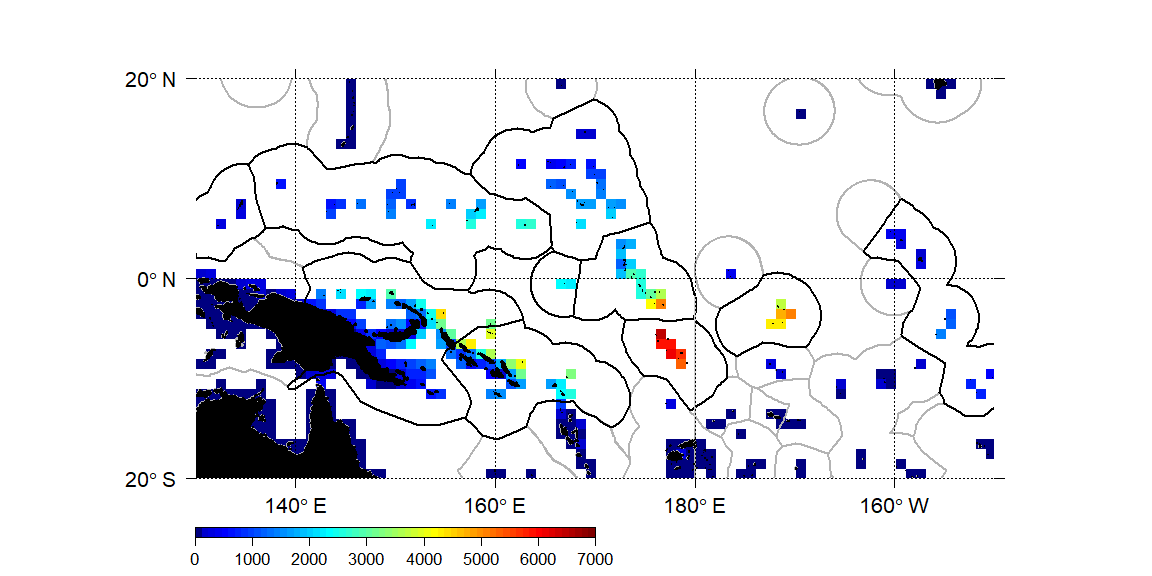


**Supplementary Figure S2.** Density of daily observed dFADs drifting through 1° land cell over 2016–2017.


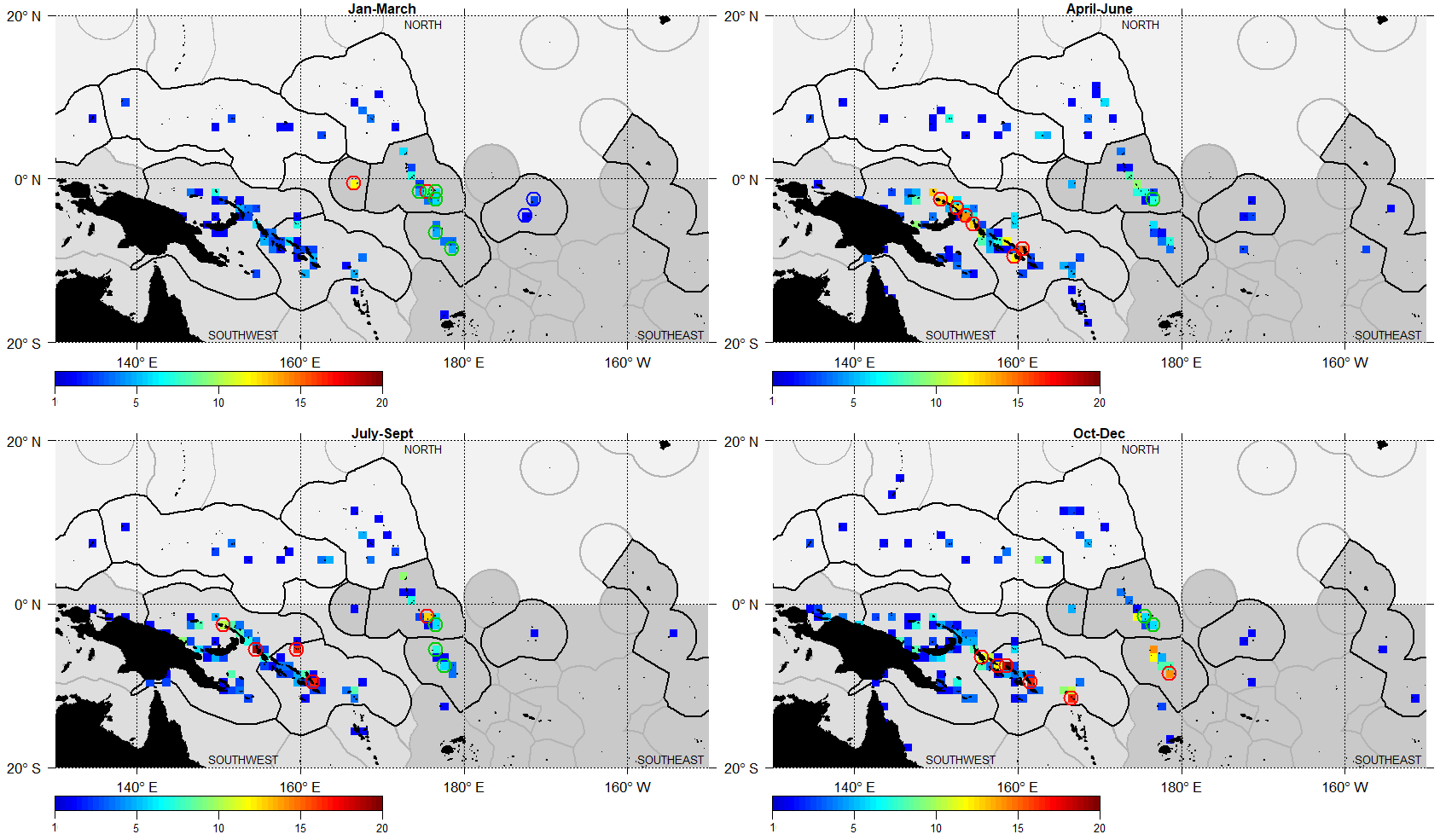


**Supplementary Figure S3.** Density of beaching events per quarter, at 1° grid cell resolution, with only coastal cells with at least one beaching represented. EEZs of countries that form the Parties to the Nauru Agreement are indicated with a solid black line. Circles represent notable beaching cells: i) high density cells (green) with high number of beaching events and high local dFAD density; ii) sensitive cells (red) with high number of beaching events but low local dFAD density; and iii) resilient cells (blue) cells with low number of beaching events and high local dFAD density.


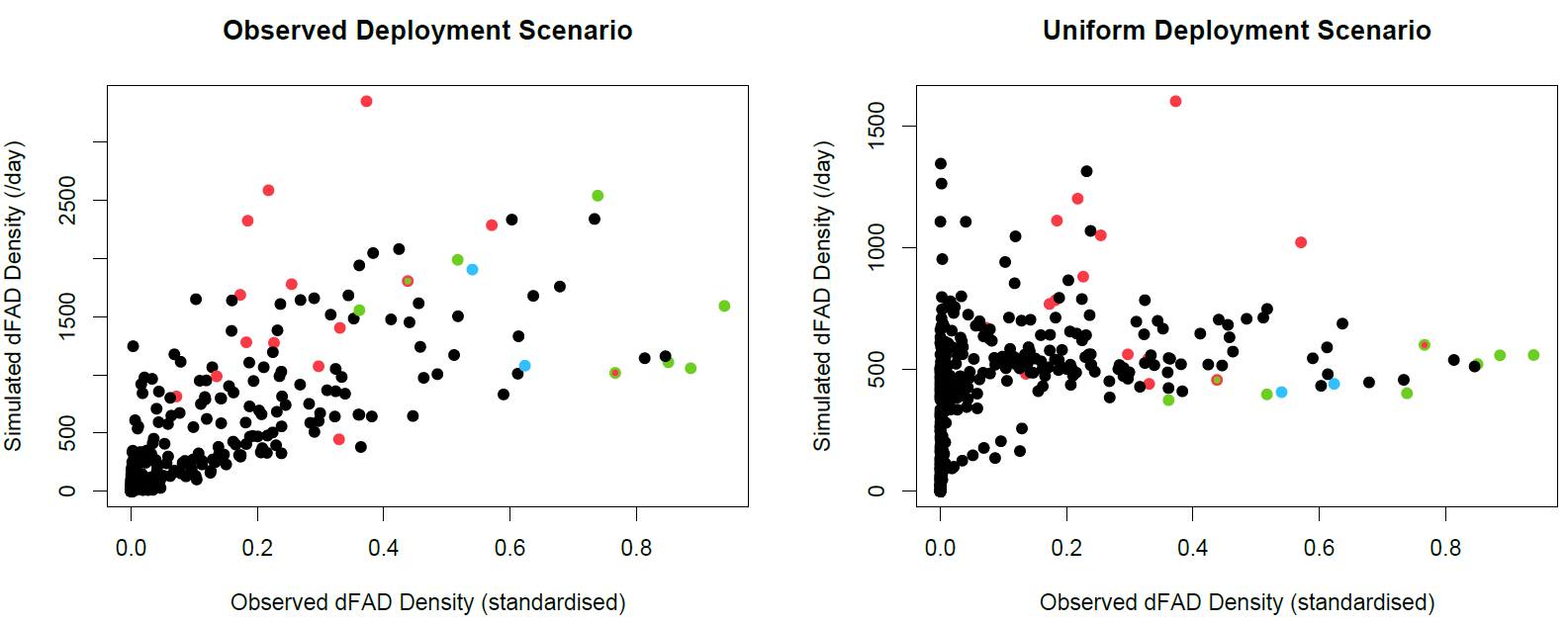


a)

b)

**Supplementary Figure S4.**  Relation between observed dFAD density in land cells and simulated dFAD density standardised by number of day for the two scenarios of a) observed deployment distribution and b) uniform deployment distribution. Coloured dots represent notable beaching cells: i) high density cells (green) with high number of beaching events and high local dFAD density; ii) sensitive cells (red) with high number of beaching events but low local dFAD density; and iii) resilient cells (blue) cells with low number of beaching events and high local dFAD density.

**
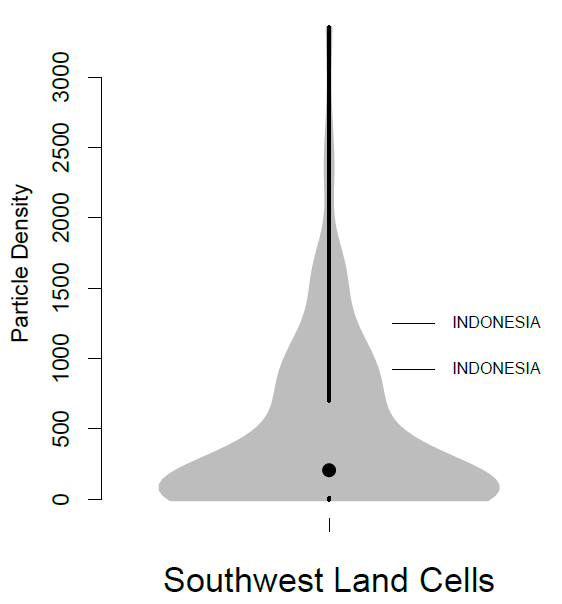
**

**Supplementary Figure S5.**  Violin plots showing the distribution of mean number of simulated particles present in each land cell each day in the southwest beaching area, with cells from non-PNA EEZs found in the top quartile of the distribution indicated on the plot. No non-PNA EEZs cells were found in the top quartile for the two other regions.
